# Direct Stimulatory Effects of the CB_2_ Ligand JTE 907 in Human and Mouse Islets

**DOI:** 10.3390/cells10030700

**Published:** 2021-03-22

**Authors:** Inmaculada Ruz-Maldonado, Patricio Atanes, Guo Cai Huang, Bo Liu, Shanta J Persaud

**Affiliations:** Department of Diabetes, School of Life Course Sciences, King’s College London, Guy’s Campus, London SE1 1UL, UK; patricio.atanes_juiz@kcl.ac.uk (P.A.); guo.huang@kcl.ac.uk (G.C.H.); bo.2.liu@kcl.ac.uk (B.L.)

**Keywords:** human islets, insulin secretion, apoptosis, proliferation, cannabinoid G protein-coupled receptors

## Abstract

Aims: The endocannabinoid system is a complex cell-signaling network through which endogenous cannabinoid ligands regulate cell function by interaction with CB_1_ and CB_2_ cannabinoid receptors, and with the novel cannabinoid receptor GPR55. CB_1_, CB_2_, and GPR55 are expressed by islet β-cells where they modulate insulin secretion. We have previously shown that administration of the putative CB_2_ antagonist/inverse agonist JTE 907 to human islets did not affect the insulinotropic actions of CB_2_ agonists and it unexpectedly stimulated insulin secretion on its own. In this study, we evaluated whether the lack of antagonism could be related to the ability of JTE 907 to act as a GPR55 agonist. Materials and Methods: We used islets isolated from human donors and from *Gpr55^+/+^* and *Gpr55^−/−^* mice and quantified the effects of incubation with 10 μM JTE 907 on dynamic insulin secretion, apoptosis, and β-cell proliferation by radioimmunoassay, luminescence caspase 3/7 activity, and immunofluorescence, respectively. We also measured islet IP_1_ and cAMP accumulation using fluorescence assays, and monitored [Ca^2+^]_i_ elevations by Fura-2 single cell microfluorometry. Results: JTE 907 significantly stimulated insulin secretion from islets isolated from human donors and islets from *Gpr55^+/+^* and *Gpr55^−/−^* mice. These stimulatory effects were accompanied by significant elevations of IP_1_ and [Ca^2+^]_i_, but there were no changes in cAMP generation. JTE 907 also significantly reduced cytokine-induced apoptosis in human and mouse islets and promoted human β-cell proliferation. Conclusion: Our observations show for the first time that JTE 907 acts as a G_q_-coupled agonist in islets to stimulate insulin secretion and maintain β-cell mass in a GPR55-independent fashion.

## 1. Introduction

Islets of Langerhans contain specialised β-cells that are capable of synthesising and secreting insulin into the bloodstream to rapidly counteract high blood glucose levels. Failure of appropriate β-cell function causes type 2 diabetes (T2D), a chronic disease that results in severe hyperglycaemia [1]. The mechanisms underlying the regulation of β-cell function and mass are of major research interest as advances in understanding will underpin development of novel pharmacological therapies for T2D [2]. In this context, the endocannabinoid system (ECS) is of particular interest since it was targeted by the cannabinoid (CB) receptor type 1 (CB_1_) antagonist rimonabant to aid weight loss and improve glycaemic control [3,4,5]. The ECS is a cell-signaling network formed by endogenous ligands, named endocannabinoids, their enzymes of synthesis and degradation, and their receptors [6,7,8,9]. Phospholipid-derived endocannabinoids such as 2-arachidonoylglycerol and anandamide act as activators of the canonical CB_1_ and CB_2_ receptors [6]. Both receptors belong to the seven-transmembrane G protein-coupled receptor (GPCR) conserved family and they are distributed centrally and peripherally in active metabolic tissues [6,9].

Several studies have demonstrated that islets express elements of the ECS [6,8,10,11,12,13,14]. Furthermore, there are reports that cannabinoid receptor ligands can regulate insulin secretion and cell viability [9,11,12,13,14,15,16,17], which are the desired pathways to be enhanced under conditions of impaired glucose tolerance or T2D [1]. However, a growing number of studies concerning cannabinoid ligand off-target effects highlight the complex pharmacology surrounding the ECS [9,15,18,19]. To date, use of synthetic cannabinoid ligands has indicated a lack of affinity of some of them for the main cannabinoid CB_1_/CB_2_ receptors, outlining independent cascades that involve other receptors with potential connections to the ECS [9,15,20]. Thus, other receptors, such as GPR55 (G protein-coupled receptor 55), have been implicated in mediating endocannabinoid effects [6,9,15,20] and playing regulatory roles in islet function and glucose homeostasis [9,15,20,21,22,23].

We have previously shown that administration of the CB_2_ antagonist/inverse agonist N-(1,3 benzodioxol-5-ylmethyl)-1,2-dihydro-7-methoxy-2-oxo-8-(pentyloxy)-3-quinolinecar boxamide, also named JTE 907 (Supplementary Figure S1), to human islets failed to inhibit the insulinotropic effects of CB_2_ agonists, but JTE 907 alone stimulated insulin secretion [10]. The stimulatory mode of action of JTE 907 in islets is unknown, so in the current study, we evaluated whether the increase in insulin secretion could be related to the ability of JTE 907 to activate the novel cannabinoid receptor GPR55. We therefore examined the functional effects of JTE 907 on insulin secretion, apoptosis, and β-cell proliferation and cAMP, IP_1_, and [Ca^2+^]_i_ signaling in islets isolated from wild-type and GPR55 null mice, and from human donors.

## 2. Materials and Methods

### 2.1. Materials

Culture media and supplements, collagenase type XI, histopaque-1077, DMSO, EDTA, IBMX, carbachol, clonidine, Accutase, Fura-2 AM, tolbutamide, LiCl, exendin-4, forskolin, agarose, bionic buffer, and BSA were obtained from Sigma-Aldrich (Dorset, UK). JTE 907 was from Tocris Bioscience (Abingdon, UK). Rabbit anti-Ki67 primary antibody was from Abcam (Cambridge, UK). cAMP HiRange and IP-one (IP_1_) assays were from Cisbio (Codolet, France). HEPES, HBSS, and DAPI were from Thermo Fisher Scientific (Paisley, UK). Caspase-Glo 3/7 was from Promega (Southampton, UK). Recombinant TNFα, IFNγ, and IL-1β were from PeproTech EC (London, UK). Guinea pig anti-insulin was purchased from Dako (Cambridge, UK). Alexa Fluor 488- and Alexa Fluor 594-conjugated secondary antibodies were from Jackson ImmunoResearch Laboratories (Newmarket, UK).

### 2.2. Animals

*Gpr55* knockout (*Gpr55^−/−^*) mice were generated by homologous recombination, with a loxP-flanked Neo cassette replacing a single GPR55 coding exon and backcrossed for 11 generations towards C57BL/6J. Mice heterozygous for the deletion were inter-crossed to produce homozygous C57BL/6J *Gpr55^−/−^* offspring [24]. The success of this approach was verified by Western blotting [22] and confirmed by PCR-based genotyping using specific primers [9]. The *Gpr55*^−/−^ mouse colony was maintained at King’s College London and provided with water and food ad libitum. Age-matched wild-type (*Gpr55^+/+^*) male C57BL/6J mice were purchased from Envigo (Bicester, UK) and maintained in the same conditions as the *Gpr55^−/−^* mice prior to islet isolation. All animal procedures were approved by the King’s College London Ethics Committee and carried out in accordance with the UK Home Office *Animals (Scientific Procedures) Act 1986* (Project Licence number PBCFBE464: Improving therapies for diabetes).

### 2.3. Isolation of Human and Mouse Islets

Human islets were isolated from 11 non-diabetic (Supplementary Table S1), heart-beating pancreas donors (3 male, 8 female) at the King’s College Hospital Islet Transplantation Unit [25], with appropriate ethical approval (KCL Human Islet Research Tissue Bank, IRAS project ID: 244510). The average age (±SEM) of the donors was 41.7 ± 3.6 years and the body mass index (BMI) was 27.8 ± 1.7 kg/m[2]. Islets were isolated from 8- to 12-week old male *Gpr55^−/−^* C57BL/6J mice and age-matched *Gpr55^+^/^+^* mice via collagenase injection into the common bile duct [26], yielding ~350 islets per mouse. Isolated mouse and human islets were maintained in culture overnight (human: CMRL-1066; mouse: RPMI-1640) at 37 °C, 95% air/5% CO_2_ prior to experimentation [27].

### 2.4. Dynamic Insulin Secretion

Groups of 55 human or 45 mouse islets were perifused with a physiological salt solution [28] supplemented with 2 mM or 20 mM glucose in the absence or presence of 10 µM JTE 907 using a temperature-controlled perifusion system [27]. Perifusate fractions were collected at 2-min intervals and secreted insulin was quantified by radioimmunoassay [26]. JTE 907 was dissolved to 10 µM in DMSO with a final DMSO concentration of 0.1% (*v*/*v*), which was also used as a control (vehicle) in the perifusions with human islets.

### 2.5. Caspase 3/7 Activities

Groups of 5 mouse or human islets were maintained in culture for 24 h in the absence or presence of 10 μM JTE 907, then incubated for a further 20 h in RPMI-1640 with 2% (*v*/*v*) FBS (mouse) or CMRL with 0.2% (*v*/*v*) albumin (human), supplemented with a cytokine cocktail (0.025 U/μL IL-1β, 1 U/μL TNFα, and 1 U/μL IFNγ). Pro-apoptotic activity was determined using the Caspase-Glo 3/7 assay [26,27].

### 2.6. Islet β-Cell Proliferation

Groups of 250 mouse or human islets were incubated for 48 h at 37 °C (95% air/5% CO_2_) in RPMI-1640 with 2% (*v*/*v*) FBS (mouse) or CMRL with 0.2% (*v*/*v*) albumin (human), supplemented with 10 JTE 907 or vehicle (0.0001% *v/v* DMSO). Human islets were also incubated with 20 nM exendin-4 as a positive control. Islets were fixed with 4% (*w*/*v*) paraformaldehyde and embedded in paraffin. Sections of 5 μm thickness were dewaxed, then antigens were retrieved using citrate buffer (10 mM citric acid, 0.05% *v/v* Tween 20, pH 6.0). For double immunofluorescence, sections were first incubated overnight at 4 °C with primary anti-insulin (guinea pig) and anti-Ki67 (rabbit) antibodies at 1:200 dilution followed by the corresponding Alexa Fluor 488/594 secondary antibodies (1:150 dilution) for 1 h at room temperature [9]. The primary and secondary antibodies used are listed in Supplementary Table S2. Human islet sections were examined under a confocal laser microscope (Nikon A1 Inverted) and mouse samples were visualised using a fluorescence microscope (Nikon Eclipse TE2000-U). Images were analysed blindly before quantification using Fiji Image J software (https://fiji.sc) [9,15]. Proliferating β-cells were quantified by counting the number of cells double-stained with Ki67 and insulin; the number of β-cells per islet was quantified by counting the number of insulin-positive cells and islet area was calculated by selecting islets with the Image J freehand selections tool and setting the scale according to the microscope objective used. For each experiment, the images were acquired with the same settings and histological quantifications were performed in paraffin sections that had been immunostained under the same conditions.

### 2.7. IP_1_ and Cyclic AMP Accumulation

Groups of 7 human islets or 5 mouse islets were transferred to 96-well plates in HBSS medium supplemented with 10 mM HEPES, 0.2% (*w*/*v*) BSA, and 2 mM IBMX for quantification of cAMP [26] or 50 mM LiCl for assay of IP_1_ [9]. Consequently, islets were incubated for 1 h at room temperature (cAMP) or at 37 °C (IP_1_) in the absence or presence of test agents, and islet cAMP or IP_1_ levels were quantified by measuring the fluorescence emission intensity ratio at 665/620 nm using a Pherastar FS microplate reader (BMG Labtech Ltd., Aylesbury, UK).

### 2.8. Single Cell Calcium Microfluorimetry

Mouse islets were dissociated by short-term incubation with the proteolytic enzyme Accutase. Groups of approximately 100,000 dispersed mouse islet cells were seeded onto glass coverslips, maintained in culture overnight, then loaded with 5 μM Fura-2 AM for 30 min. Cells on coverslips were perifused (37 °C, 1 mL/min) with a physiological salt solution [28] containing 2 mM glucose in the absence or presence of test agents. Real-time changes in [Ca^2+^]_i_ were determined by illuminating cells alternately at 340 nm and 380 nm, with the emitted light being filtered at 510 nm and data were recorded with a CCD camera every 3 s [26].

### 2.9. Statistical Analyses

All data were analyzed by unpaired Student’s *t*-test comparisons or one-way ANOVA with repeated measures followed by post hoc tests, as appropriate, using GraphPad Prism 8.0 (GraphPad Software, Inc.). All numerical values are presented as mean + SEM and *p* < 0.05 was considered statistically significant.

## 3. Results

### 3.1. JTE 907 Stimulates Insulin Secretion in a GPR55-Independent Manner

**Figure 1:**
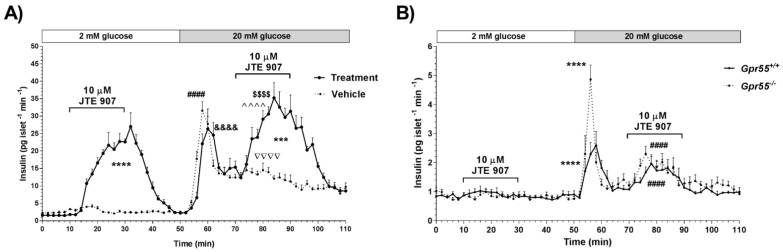
Effects of JTE 907 on dynamic insulin secretion from human and mouse islets. (**A**) Human islets: 10 µM JTE 907 significantly stimulated insulin secretion from human islets at 2 mM and 20 mM glucose compared to vehicle-treated islets shown by the dotted line. Data are expressed as mean + SEM of 4 replicates within an individual experiment, representative of 3 separate experiments, 55 islets per perifusion chamber; **** *p* < 0.0001 and *** *p* < 0.001 vs. vehicle; ^&&&&^ *p* < 0.0001 vs. min 0–10 treatment; ^^^^ *p* < 0.0001 min 70–90 vs. min 50–70 treatment; ^$$$$^ *p* < 0.0001 min 70–90 vs. min 0–10 treatment; ^####^ *p* < 0.0001 min 50–70 vs. min 0–10 vehicle; ^˅˅˅˅^ *p* < 0.0001 min 70–90 vs. min 0–10 vehicle; one-way ANOVA, Tukey’s multiple comparisons post-test. (**B**) Mouse islets: 10 µM JTE 907 also significantly potentiated glucose-stimulated insulin secretion from WT mouse islets and the effects were maintained in *Gpr55^−/−^* islets, shown by the dotted line. Data are expressed as mean + SEM of 4 replicates within an individual experiment, representative of 3 separate experiments, 45 islets per perifusion chamber; **** *p* < 0.0001 min 50–70 vs. min 0–10 (*Gpr55^+/+^* and *Gpr55^−/−^*); ^####^ *p* < 0.0001 min 70–90 vs. min 0–10 (*Gpr55^+/+^* and *Gpr55^−/−^*); two-way ANOVA, Tukey’s multiple comparisons post-test.

In an earlier study, we demonstrated that JTE 907 did not inhibit insulin secretion induced by the CB_2_ agonist JWH 015 in human islets, and it had independent stimulatory effects on insulin release that were similar to those of JWH 015 [10]. Here, we analysed dynamic insulin secretion from human and mouse islets perifused with buffers supplemented with 2 mM glucose followed by 20 mM glucose in the absence or presence of 10 µM JTE 907. Figure 1A indicates that JTE 907 induced significant, reversible elevations in insulin secretion from human islets during perifusion at both 2 mM and 20 mM glucose, compared to islets treated with vehicle alone. To evaluate whether this direct stimulation of insulin release could be related to the ability of JTE 907 to act as a GPR55 agonist in islets, we examined dynamic insulin profiles using wild-type mice and those null for GPR55, and found that JTE 907 significantly promoted glucose-stimulated insulin secretion in both *Gpr55^+/+^* and *Gpr55^−/−^* mouse islets (Figure 1B). Potentiation of glucose-induced insulin secretion from *Gpr55^−/−^* islets by JTE 907 thus ruled out the involvement of GPR55 signaling in its insulin secretagogue effects.

### 3.2. JTE 907 Protects Human and Mouse Islets from Cytokine-Induced Apoptosis

**Figure 2:**
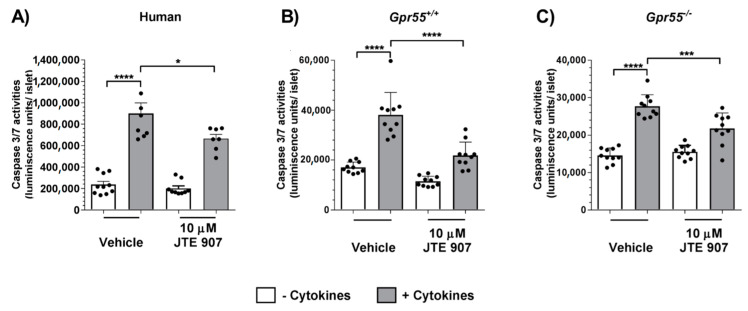
Effects of JTE 907 on human and mouse islet apoptosis. 10 μM JTE 907 significantly protected against cytokine-induced apoptosis in human (**A**) and mouse (**B**,**C**) islets, but had no significant effect on basal apoptosis. Data show mean + SEM data of 8–10 observations per treatment group that are representative of 3 independent experiments using both mouse and human islets. * *p* < 0.05, *** *p* < 0.001, **** *p* < 0.0001, *p* > 0.05 (vehicle vs. JTE 907 in the absence of cytokines). Data were analysed using one-way ANOVA, followed by Tukey’s multiple comparisons post-test.

To examine whether the insulinotropic effects of JTE 907 in human and mouse islets were accompanied by alterations in islet apoptosis, we evaluated whether exposure of islets to JTE 907 affected basal or cytokine-induced caspase 3/7 activities in islets isolated from human donors and from *Gpr55^+/+^* and *Gpr55^−/−^* mice. In these experiments, 10 µM JTE 907 significantly decreased apoptosis stimulated by cytokines in human islets (Figure 2A). Moreover, JTE 907 also inhibited cytokine-induced apoptosis in islets isolated from *Gpr55^+/+^* mice (Figure 2B), and these anti-apoptotic properties were also observed in islets from *Gpr55^−/−^* mice (Figure 2C). However, although this ligand had protective effects against cytokines, it did not significantly reduce basal levels of apoptosis in the absence of cytokines in either human or mouse islets.

### 3.3. JTE 907 Induces Human β-Cell Proliferation

**Figure 3:**
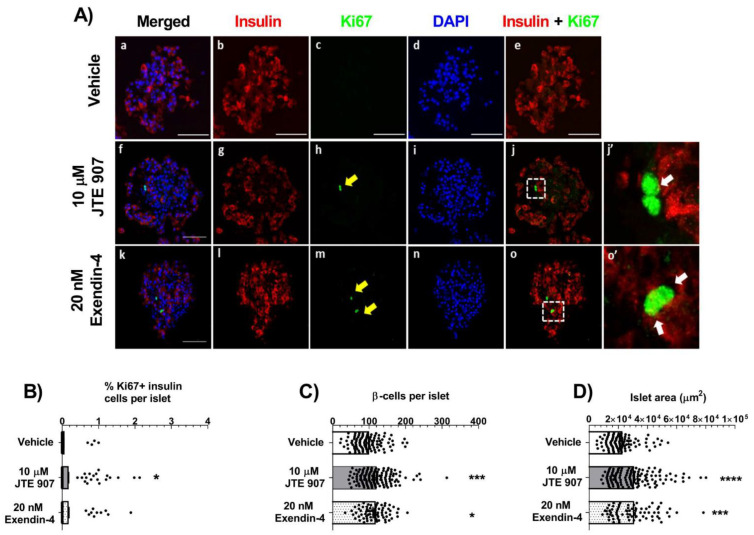
Effects of JTE 907 on human β-cell proliferation. Representative confocal images of paraffin-embedded sections of human islets from a single donor probed with antibodies directed against insulin (red) and Ki67 (green), and DAPI staining (nuclei; blue) after maintenance of islets in culture for 48 h in the absence or presence of 10 µM JTE 907 and exendin-4 (**A**). Scale bars = 50 μm. White arrows in panels j’ and o’ denote proliferating β-cells. Quantifications (**B**–**D**) were performed using islets from 3 separate donors and were obtained from 3 acquisitions of 122– 153 islets per condition, each with a minimum of 40 different islets analysed per paraffin section. * *p* < 0.05, *** *p* < 0.001, **** *p* < 0.0001 vs. vehicle. Data were analysed using one-way ANOVA, followed by Dunnett’s multiple comparison post-test. Analysis of mean data from 3 donors indicated that 10 µM JTE 907 increased the % Ki67-positive β-cells (**B**), compared to vehicle-treated islets, and it also significantly increased the number of β-cells (insulin-positive cells) per islet (**C**) and islet area (**D**). These stimulatory effects of JTE 907 were similar to those observed with the GLP-1 analogue exendin-4.

**Figure 4:**
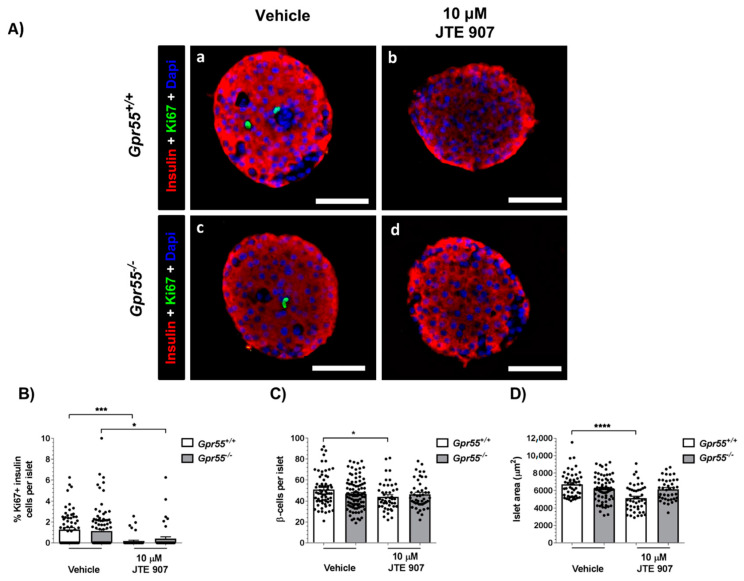
Effects of JTE 907 on mouse β-cell proliferation. Representative fluorescence images of paraffin-embedded sections of islets from individual *Gpr55^+/+^* and *Gpr55^−/−^* mice probed with antibodies directed against insulin (red) and Ki67 (green), and DAPI staining (nuclei; blue) after maintenance of islets in culture for 48 h in the absence or presence of 10 µM JTE 907 (**A**). Scale bars = 50 μm. Quantifications (**B**–**D**) were performed using islets from 6 mice per genotype and were obtained from 3 acquisitions of 49–97 islets per condition, each with a minimum of 16 different islets analysed per paraffin section. * *p* < 0.05, *** *p* < 0.001, **** *p* < 0.0001. Data were analysed using one-way ANOVA, followed by Tukey’s multiple comparisons post-test.

The role of the JTE 907 in regulating adult β cell-proliferation was investigated by co-staining of insulin and the proliferative marker Ki67 in human and mouse isolated islets. Mean analysis of islets from three separate donors revealed that exposure to 10 µM JTE 907 for 48 h significantly increased the small percentage of insulin-positive cells expressing Ki67 (Figure 3A,B). This increased human β-cell proliferation was associated with small increases in the number of β-cells per islet (Figure 3C) and in islet area (Figure 3D). The GLP-1 analogue, exendin-4, also significantly stimulated human islet β-cell proliferation (Figure 3A,B) and increased β-cell number (Figure 3C) and islet area (Figure 3D). When the effects of JTE 907 were analysed in islets from individual donors, it was found that it had pro-proliferative effects in two batches of islets but not in the third (Supplementary Figure S2), suggesting that the ability of JTE 907 to stimulate human β-cell proliferation is variable. In contrast to the data obtained with human islets, the β-cell proliferation rates in vehicle-treated *Gpr55^+/+^* and *Gpr55^−/−^* mouse islets were significantly decreased after exposure to 10 μM JTE 907 for 48 h (Figure 4A,B). Regarding β-cell mass, 10 μM JTE 907 also had a small impact, reducing the number of β-cells per islet (Figure 4C) and islet area (Figure 4D) in islets isolated from *Gpr55^+/+^* mice. However, no significant changes in the number of β-cells per islet (Figure 4C) or islet area (Figure 4D) were identified in *Gpr55^−/−^* islets following 48 h exposure to 10 μM JTE 907.

### 3.4. JTE 907 Is Not Coupled to G_s_ or G_i_ Signaling in Human and Mouse Islets

**Figure 5:**
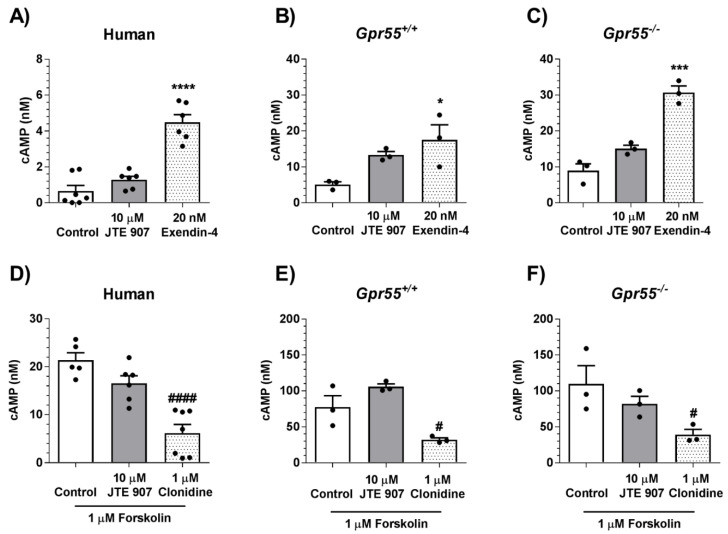
Effects of JTE 907 on cAMP levels in human and mouse islets. 10 μM JTE 907 had no effect on cAMP accumulation in untreated human islets (**A**) or those exposed to 1 μM forskolin (**D**). JTE 907 also did not significantly affect cAMP levels in islets isolated from *Gpr55^+/+^* (**B**,**E**) and *Gpr55^−/−^* (**C**,**F**) mice (*p* > 0.05 for all JTE 907 vs. control comparisons). In the same experiments, 20 nM exendin-4 and 1 μM clonidine significantly increased and decreased cAMP, respectively (**A**–**F**). Data show mean + SEM data of 3–6 observations per treatment group that are representative of 3 independent experiments using both mouse and human islets. * *p* < 0.05, *** *p* < 0.001, and **** *p* < 0.0001 exendin-4 vs. basal; ^#^ *p* < 0.05 and ^####^ *p* < 0.0001 clonidine vs. forskolin. Data were analysed using one-way ANOVA, followed by Dunnett’s multiple comparisons post-test.

In an attempt to delineate the downstream mechanisms activated by JTE 907 in human and mouse islets, we measured cAMP accumulation to examine whether it was coupled to G_s_ signaling. However, 10 µM JTE 907 did not significantly stimulate cAMP elevations in human islets nor islets isolated from *Gpr55^+/+^* and *Gpr55^−/−^* mice (*p* > 0.05 for all comparisons), whereas the GLP-1 analogue exendin-4 (20 nM) significantly increased cAMP accumulation, as expected (Figure 5A–C). In human (Figure 5D) and mouse islets (Figure 5E,F), 10 µM JTE 907 also failed to significantly inhibit forskolin-elevated cAMP accumulation, confirming that it does not couple to G_i_ or signal via GPR55. As expected, the α_2_-adrenergic agonist clonidine significantly inhibited forskolin-induced elevation in cAMP in both human and mouse islets (Figure 5D–F).

### 3.5. JTE 907 Increases Islet IP_1_ Levels in Human and Mouse Islets

**Figure 6:**
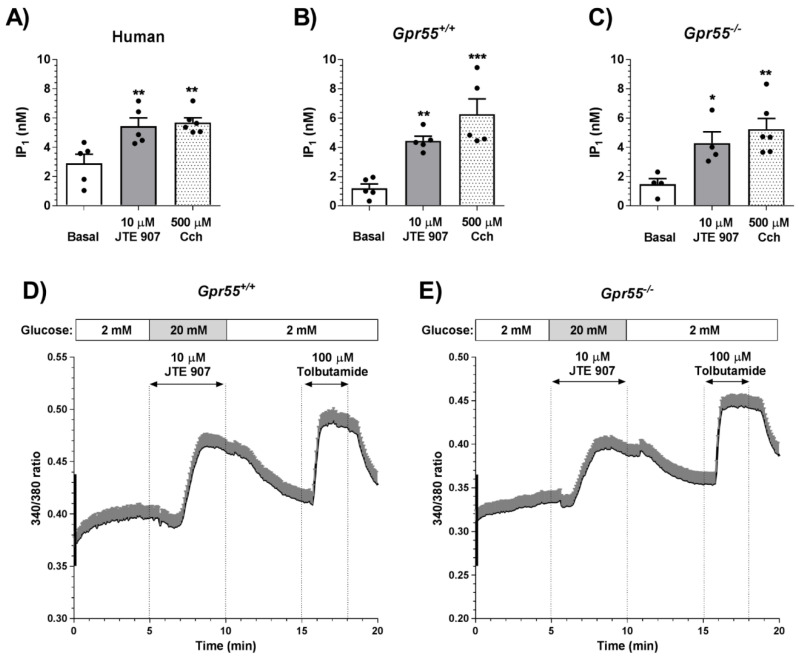
Effects of JTE 907 on IP_1_ levels and [Ca^2+^]_i_ in islets. (**A**–**C**) 10 μM JTE 907 significantly elevated IP_1_ levels in islets from human donors (**A**) and *Gpr55^+/+^* (**B**) and *Gpr55^−/−^* (**C**) mice. 500 μM carbachol (Cch) was used as positive control. Panels A–C show mean + SEM data of 4–6 observations per treatment group that are representative of 3 independent experiments using both mouse and human islets. * *p* < 0.05, ** *p* < 0.01, *** *p* < 0.001 JTE 907 or Cch vs. basal. Data were analysed using one-way ANOVA, followed by Dunnett’s multiple comparisons post-test. (**D**,**E**) Dynamic profiles of [Ca^2+^]_i_ in Fura-2-loaded dispersed *Gpr55^+/+^* (**D**) or *Gpr55^−/−^* (**E**) mouse islet cells. 10 µM JTE 907 induced [Ca^2+^]_i_ elevation in the presence of 20 mM glucose in both *Gpr55^+/+^* and *Gpr55^−/−^* islet cells. The horizontal arrows indicate the period of perifusion with JTE 907 or the sulphonylurea tolbutamide, which was used as a positive control. Data of the 340/380 fluorescence ratios are expressed as mean + SEM of 68 (**D**) and 74 (**E**) islet cells from 3 independent experiments with *Gpr55^+/+^* or *Gpr55^−/−^* mouse islets.

We also determined the effects of JTE 907 on G_q_ coupling in islets by quantifying levels of the stable IP_3_ metabolite, IP_1_. In human (Figure 6A) and mouse (Figure 6B,C) islets, 10 µM JTE 907 significantly increased IP_1_ levels, as did the muscarinic receptor agonist carbachol, which signals via G_q_-coupled M_3_ receptors in islets [29]. JTE 907 also significantly elevated IP_1_ in islets in which GPR55 had been deleted (Figure 6C), consistent with the maintenance of its stimulation of insulin secretion and protection against cytokine-induced apoptosis in islets from *Gpr55^−/−^* mice. In parallel experiments, we performed single cell calcium microfluorimetry measurements in Fura-2-loaded mouse islet cells. These experiments indicated that 10 µM JTE 907 elevated [Ca^2+^]_i_ in islet cells from both *Gpr55^+/+^* (Figure 6D) and *Gpr55^−/−^* (Figure 6E) mice at 20 mM glucose, in agreement with the islet IP_1_ measurements. In all tested batches of islets, the ATP-sensitive K^+^ channel blocker, tolbutamide, stimulated reversible increases in [Ca^2+^]_i_, demonstrating that the ability to control the membrane potential was preserved after exposure to JTE 907.

## 4. Discussion

There are many studies indicating that effects of cannabinoid ligands are not limited to the classical CB_1_ and CB_2_ receptors, and it has been suggested that off-target effects of cannabinoids may be mediated by the novel cannabinoid receptor GPR55 [30]. We, and others, have shown that GPR55 is expressed by mouse and human β-cells where it is coupled to the stimulation of insulin secretion [15,21,22]. We have previously reported that JTE 907, a CB_2_ antagonist/inverse agonist, unexpectedly had direct insulinotropic effects in human islets when it was administered alone, similar to those of the CB_2_ agonist JWH 015 [10]. In the current study, we evaluated whether JTE 907 acted through GPR55-dependent coupling in islets to promote insulin secretion, and we also investigated its effects on human and mouse β-cell mass.

We identified that JTE 907 reversibly stimulated insulin output from isolated human and mouse islets, confirming our earlier observation of insulinotropic effects induced by JTE 907 in dynamic secretion experiments with human islets [10]. JTE 907 also evoked significant stimulation of insulin from human islets at 2 mM glucose, but had no effects in mouse islets under these hypoglycaemic conditions. The capacity of JTE 907 to initiate insulin secretion from human islets at sub-stimulatory glucose indicates that this drug would not be suitable for in vivo delivery as there would be the potential risks of hypoglycaemic episodes with its use. The reasons underlying the disparity in the glucose-dependent effects of JTE 907 between human and mouse islets are unclear, but it could be a consequence of inter-species differences in islet architecture, where human islets contain proportionally more α-cells than do mouse islets [31,32], or it could reflect species-dependent differences in metabolic requirements [33,34]. It is also possible that JTE 907 has more than one mode of action, and the mechanism through which it elevates basal insulin secretion is present in human, but not mouse, islets while the potentiating pathway is common to both. Further research is required to fully define signaling downstream of JTE 907 in mouse and human islets, as discussed below. Nonetheless, the maintenance of the stimulatory effects of JTE 907 in perifused islets isolated from *Gpr55^−/−^* mice demonstrated that its ability to induce insulin secretion is not dependent on GPR55 activation.

In the current study, we also showed that, in addition to stimulating insulin secretion, JTE 907 has direct anti-apoptotic effects in isolated human islets. It is possible that, although it does not signal via GPR55 in islets to increase insulin secretion, JTE 907 could activate this receptor to protect β-cells against cytokine-induced apoptosis. Thus, it is known that GPR55 activation protects against cytokine-induced apoptosis in human islets [15] and it has anti-apoptotic, anti-inflammatory and cytoprotective effects in mouse islets [15,20]. However, although JTE 907 also reduced cytokine-induced caspase 3/7 activities in mouse islets, the use of islets from *Gpr55^−/−^* mice indicated that, as for stimulation of insulin secretion, this was through a GPR55-independent cascade.

Immunohistochemical analyses of Ki67 and insulin staining indicated that JTE 907 significantly increased the low rate of adult human β-cell proliferation, to a similar extent to that seen with the GLP-1 analogue exendin-4. We have previously demonstrated that the putative CB_1_ antagonist LH-21 acted as a GPR55 agonist to induce mouse β-cell proliferation [15]. In the current study, we therefore expanded the investigation into the potential effect of JTE 907 on mouse β-cell proliferative activity and determined whether this ligand mediated its actions via GPR55. However, in contrast to the data obtained with human islets, JTE 907 reduced the mouse β-cell proliferation index in islets isolated from *Gpr55^+/+^* mice, and this was associated with small decreases in the number of β-cells per islet and islet area. A similar JTE 907-induced inhibition of β-cell proliferation was observed in islets from *Gpr55^−/−^* mice, but β-cell number or islet area were not affected. It is unlikely that this reflects GPR55-dependent effects of JTE 907 since it inhibited β-cell proliferation to a similar extent in *Gpr55^+/+^* and *Gpr55^−/−^* islets. This suggests that the balance of decreased proliferation and decreased apoptosis that we observed in response to JTE 907 resulted in an overall lack of effect on β-cell number or islet area. The reasons underlying the differences in the proliferative effects of JTE 907 in human and mouse islets are not immediately obvious: the protocols were standardised, but while the mice used for islet isolation in this study were male 8–12 weeks old, fed on a standard chow diet, donors of human islets for research are generally middle-aged and overweight or obese. In particular, the islets used for our proliferation experiments were from obese female donors, with mean age and BMI of 38.0 ± 2.9 and 35.5 ± 2.8, respectively (see Supplementary Table S1). It is known that β-cells exhibit better proliferation potential in obesity [35], so this may have increased the opportunity to detect elevated proliferation with JTE 907 in our experiments. Our analysis of the effects of JTE 907 on β-cell proliferation in islets from individual donors supported this, with the most marked stimulatory effects of JTE 907 observed in islets obtained from a donor with a BMI of 39.5 (Donor 10 in Supplementary Table S1, panels D–F in Supplementary Figure S2). The individual data also indicated that there are inter-donor differences in responses to both JTE 907 and exendin-4. It was not possible to define the variabilities in islet donor characteristics that could affect experimental outcomes in this study, but there may have been differences in the metabolic status of the donors or in the proportion of β-cells per islet, despite the donors being well matched for gender, age, and BMI. We did not have sufficient samples to address whether there were gender- or age-dependent effects on human β-cell proliferation in the absence and presence of JTE 907, nor whether JTE 907 also induced proliferation in islets from lean donors, and these are experiments that would be useful for future studies.

Our experiments indicating that JTE 907 does not antagonize CB_2_-mediated signaling in islets [10], together with our current demonstration that JTE 907 has direct effects on islet function that is maintained in islets in which GPR55 is absent, are consistent with JTE 907 having CB_2_- and GPR55-independent signaling in islets. We therefore quantified cAMP and IP_1_ levels to determine whether JTE 907 was signaling via traditional GPCR cascades in human and mouse islets. Quantification of cAMP levels indicated that JTE 907 did not act via G_s_ to transduce adenylate cyclase-mediated cAMP accumulation, demonstrating that it was not activating receptors involving stimulatory G_s_ signaling. The CB_2_ inverse agonist action of JTE 907 is associated with enhanced forskolin-stimulated cAMP generation [36], but this was not observed in islets, again confirming that JTE 907 was not acting via CB_2_ in islets. Furthermore, we ruled out an effect of JTE 907 via G_i_ activation in human and mouse islets since it did not inhibit cAMP accumulation in the presence of forskolin. This fits with the physiological effects of JTE 907 to increase insulin secretion, which would not be compatible with G_i_-coupled signaling.

JTE 907 significantly elevated IP_1_ levels in human and mouse islets implying activation of G_q_ and phospholipase C-mediated hydrolysis of phosphatidylinositol 4,5-bisphosphate to generate IP_3_ and diacylglycerol. This signaling cascade is known to be coupled to increased insulin secretion, protection against apoptosis, and stimulation of proliferation in islets [9,15,22,37], so it seems likely that the effects of JTE 907 that we have presented here are mediated by activation of a G_q_-coupled GPCR. GPR55 signals via G_q_, but, consistent with our functional data, JTE 907 did not act via GPR55 to elevate IP_1_ since its stimulatory effects were maintained in islets from *Gpr55^−/−^* mice. In addition, JTE 907 induced intracellular [Ca^2+^]_i_ elevations in islet cells from both *Gpr55^+/+^* and *Gpr55^−/−^* mice, in agreement with GPR55-independent G_q_ coupling. The receptor responsible for mediating the effects of JTE 907 in islets is unknown. There are several GPCRs that have been implicated in cannabinoid signaling: we have ruled out GPR55 in the current experiments, GPR119 is not a candidate since it is G_s_-coupled, but GPR18 and GPR92 are G_q_-coupled and can be activated by some cannabinoids [38,39]. However, we have previously reported that GPR18 is not expressed by human islets [40], so this receptor could not have been mediating the effects of JTE 907 in human islets that we report here. In addition, although GPR92 appears to be a plausible candidate, its activation is reported to lead to elevations in cAMP [41], which we did not observe here with JTE 907. Human islets express nearly 300 GPCRs [40], so it is clear that further work is required to define the GPCR responsible for the effects of JTE 907.

## 5. Conclusions

Our study, using isolated human and mouse islets, demonstrates that the CB_2_ antagonist/inverse agonist JTE 907 has direct effects to stimulate insulin secretion and preserve β-cell mass, which are independent of CB_2_ or GPR55 signaling. We have identified that JTE 907 activates G_q_ coupling in islets, but the GPCR through which it acts has not yet been identified.

## Data Availability

Data available upon request.

## Supplementary Materials

The following are available online at https://www.mdpi.com/2073-4409/10/3/700/s1, Figure S1: Chemical structure of JTE 907, Figure S2: Effects of JTE 907 on human-cell proliferation in islets from individual donors, Table S1: Human islet preparations obtained from non-diabetic donors used in this study, Table S2: List of primary and secondary antibodies used for the immunofluorescence staining methods.

## Abbreviations

BMI	body mass index
BSA	bovine serum albumin
cAMP	3′,5′-cyclic adenosine monophosphate
Cch	carbachol
CB_1_	canonical cannabinoid receptor type 1
CB_2_	canonical cannabinoid receptor type 2
CMRL	Connaught Medical Research Laboratories
DAPI	4′,6-diamidino-2-phenylindole
DMSO	dimethyl sulfoxide
ECS	endocannabinoid system
EDTA	ethylenediaminetetraacetic acid
FBS	fetal bovine serum
GLP-1	glucagon-like peptide-1
GPCR	G protein-coupled receptor
GPR55	G protein-coupled receptor 55
G_q_	G_q_ alpha subunit
G_s_	G_s_ alpha subunit
HBSS	Hank’s Balanced Salt Solution
HEPES	4-(2-hydroxyethyl)-1-piperazineethanesulfonic acid
HTRF	Homogeneous Time Resolved Fluorescence
IBMX	3-isobutyl-1-methylxanthine
IFNγ	interferon gamma
IL-1β	interleukin-1 beta
IP_1_	inositol-1-phosphate
IP_3_	inositol trisphosphate
JTE 907	N-(1,3 benzodioxol-5-ylmethyl)-1,2-dihydro-7-methoxy-2-oxo-8-(pentyloxy)-3-quinolinecar boxamide
JWH 015	(2-Methyl-1-propyl-1H-indol-3-yl)-1-naphthalenylmethanone
LH-21	5-(4-chlorophenyl)-1-(2,4-dichlorophenyl)-3-hexyl-1H-1,2,4-triazole
M_3_	muscarinic receptors
RIA	radioimmunoassay
RPMI	Roswell Park Memorial Institute
Rimonabant (SR141716A)	5-(4-chlorophenyl)-1-(2,4-dichloro-phenyl)-4-methyl-N-(piperidin1-yl)-1H-pyrazole-3-carboxamide
TNF_α_	tumor necrosis factor alpha
T2D	type 2 diabetes
