# Supplementary material for: Direct Stimulatory Effects of the CB2 Ligand JTE 907 in Human and Mouse Islets
Source: Cells. 2021 Mar 22;10(3):700. doi: 10.3390/cells10030700 (PMC8004177; doi:10.3390/cells10030700)
Supplement: Supplementary file 1 [file cells-10-00700-s001.pdf]

SUPPLEMENTARY DATA

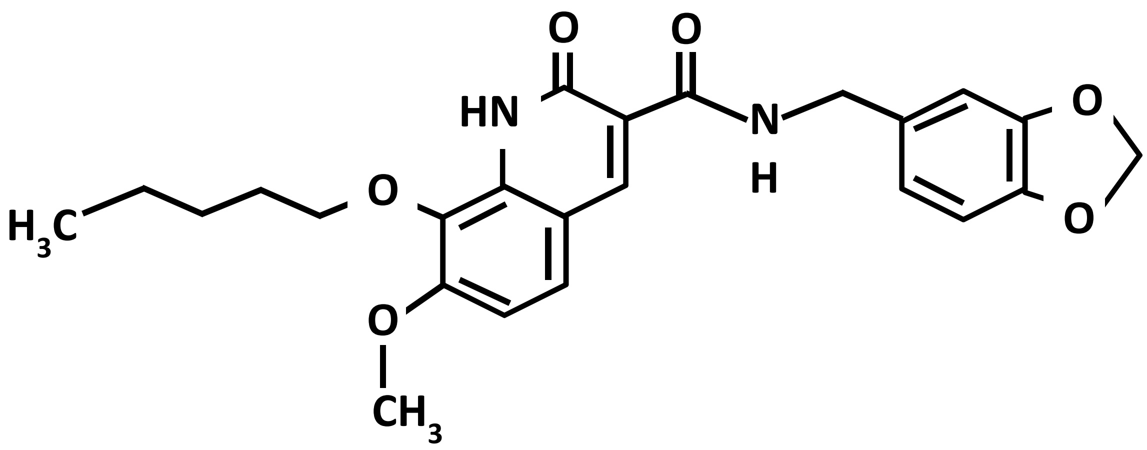

Figure S1. Chemical structure of JTE 907.

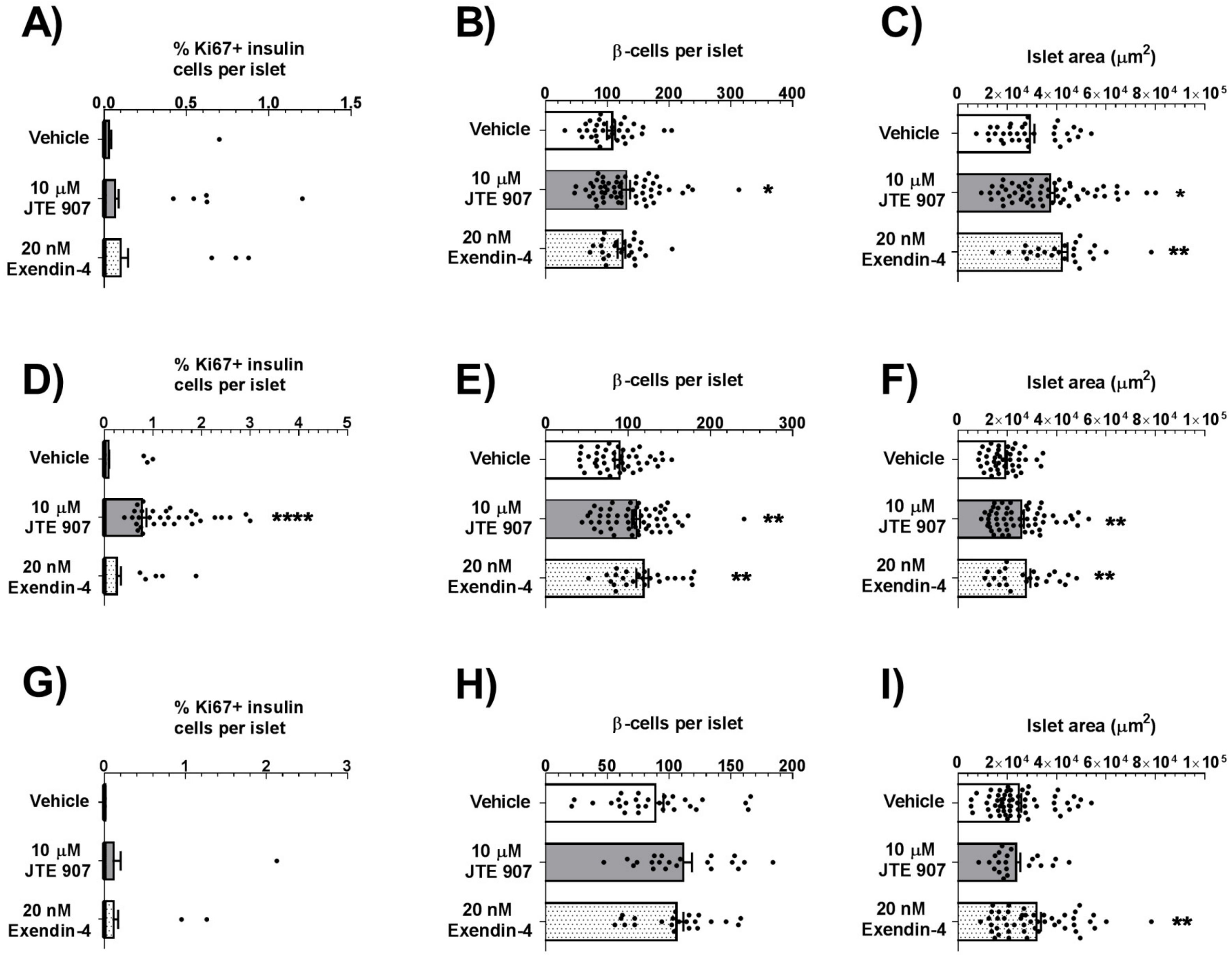

A)
% Ki67+ insulin cells per islet
0.0
0.5
1.0
1.5
Vehicle
10 μM JTE 907
20 nM Exendin-4
B)
β-cells per islet
0
100
200
300
400
Vehicle
10 μM JTE 907
20 nM Exendin-4
*
C)
Islet area (μm2)
0
2×10^4
4×10^4
6×10^4
8×10^4
1×10^5
Vehicle
10 μM JTE 907
20 nM Exendin-4
*
**
D)
% Ki67+ insulin cells per islet
0
1
2
3
4
5
Vehicle
10 μM JTE 907
20 nM Exendin-4
****
E)
β-cells per islet
0
100
200
300
Vehicle
10 μM JTE 907
20 nM Exendin-4
**
**
F)
Islet area (μm2)
0
2×10^4
4×10^4
6×10^4
8×10^4
1×10^5
Vehicle
10 μM JTE 907
20 nM Exendin-4
**
**
G)
% Ki67+ insulin cells per islet
0
1
2
3
Vehicle
10 μM JTE 907
20 nM Exendin-4
H)
β-cells per islet
0
50
100
150
200
Vehicle
10 μM JTE 907
20 nM Exendin-4
I)
Islet area (μm2)
0
2×10^4
4×10^4
6×10^4
8×10^4
1×10^5
Vehicle
10 μM JTE 907
20 nM Exendin-4
**

SUPPLEMENTARY Figure S2. Effects of JTE 907 on human β-cell proliferation in islets from individual donors.

Panels A-I show the proliferation data analysis in human islets from individual donors. The data shown in panels A-C, D-F and G-I were obtained from donors 4, 10 and 2, respectively (see Supplementary Table 1). $^{*}p < 0.05$, $^{**}p < 0.01$, $^{****}p < 0.0001$ *vs.* vehicle. Data were analysed using one-way ANOVA, followed by Dunnett's multiple comparison post test. The mean data are shown in Figure 3.

SUPPLEMENTARY Table S1. Human islet preparations obtained from non-diabetic donors used in this study.

Donor	Gender	Age (Years)	BMI (kg/m^2)	Human Islet Donor Used Per Figure
Number 1	Female	23	20	Figure 2A
Number 2	Female	43	30	Figures 1, 3, Figure S2
Number 3	Male	34	26	Figure 1
Number 4	Female	38	37	Figure 3, Figure S2
Number 5	Male	60	32	Figure 2A
Number 6	Female	37	30	Figures 5A, 5D
Number 7	Female	40	27	Figure 1
Number 8	Female	53	23	Figures 5A, 5D, 6A
Number 9	Female	45	31	Figures 2A, 6A
Number 10	Female	33	39.5	Figures 3, 5A, 5D, Figure S2
Number 11	Male	53	22	Figure 6A

SUPPLEMENTARY Table S2. List of primary and secondary antibodies used for the immunofluorescence staining methods.

Antibody	Dilution	Vendor	Catalogue Number
Anti-Ki67 (rabbit)	1:200	Abcam	ab15580
Anti-insulin (guinea pig)	1:200	Dako	A0564
AlexaFluor 488 (goat anti-rabbit)	1:150	Jackson ImmunoResearch Laboratories	111-545-003
AlexaFluor 594 (donkey anti-guinea pig)	1:150	Jackson ImmunoResearch Laboratories	711-586-152
